# Ecological traps in shallow coastal waters—Potential effect of heat-waves in tropical and temperate organisms

**DOI:** 10.1371/journal.pone.0192700

**Published:** 2018-02-08

**Authors:** Catarina Vinagre, Vanessa Mendonça, Rui Cereja, Francisca Abreu-Afonso, Marta Dias, Damián Mizrahi, Augusto A. V. Flores

**Affiliations:** 1 MARE–Marine and Environmental Sciences Centre, Universidade de Lisboa, Faculdade de Ciências, Campo Grande, Lisboa, Portugal; 2 Centro de Biologia Marinha, Universidade de São Paulo, Rod. Manoel Hipólito do Rego, São Sebastião, SP, Brazil; Universidad de la Republica Uruguay, URUGUAY

## Abstract

Mortality of fish has been reported in tide pools during warm days. That means that tide pools are potential ecological traps for coastal organisms, which happen when environmental changes cause maladaptive habitat selection. Heat-waves are predicted to increase in intensity, duration and frequency, making it relevant to investigate the role of tide pools as traps for coastal organisms. However, heat waves can also lead to acclimatization. If organisms undergo acclimatization prior to being trapped in tide pools, their survival chances may increase. Common tide pool species (46 species in total) were collected at a tropical and a temperate area and their upper thermal limits estimated. They were maintained for 10 days at their mean summer sea surface temperature +3°C, mimicking a heat-wave. Their upper thermal limits were estimated again, after this acclimation period, to calculate each species’ acclimation response. The upper thermal limits of the organisms were compared to the temperatures attained by tide pool waters to investigate if 1) tide pools could be considered ecological traps and 2) if the increase in upper thermal limits elicited by the acclimation period could make the organisms less vulnerable to this threat. Tropical tide pools were found to be ecological traps for an important number of common coastal species, given that they can attain temperatures higher than the upper thermal limits of most of those species. Tide pools are not ecological traps in temperate zones. Tropical species have higher thermal limits than temperate species, but lower acclimation response, that does not allow them to survive the maximum habitat temperature of tropical tide pools. This way, tropical coastal organisms seem to be, not only more vulnerable to climate warming *per se*, but also to an increase in the ecological trap effect of tide pools.

## Introduction

Ecological traps, which happen when environmental changes cause maladaptive habitat selection, have been identified for many species, in diverse ecosystems, most of them terrestrial [1–3]. They are likely to increase local extinction risk, thus understanding where they may occur and what species will be affected is important for conservation biology. Global warming has the potential to dramatically change environments worldwide. These changes should occur at rapid rates exposing animals to conditions they have not experienced in their evolutionary history.

Shallow coastal waters are among the habitats where the impacts of climate warming will be apparent more rapidly, making these areas useful natural laboratories, not only for the study of community dynamics as they traditionally have been, but also for climate change research [4–11]. Their low depth means that they have a lower thermal inertia than open-ocean waters, and that their thermal regime is affected by both oceanographic and atmospheric conditions. In fact, such habitats have been considered early warning systems for climate change impacts [4]. These shallow coastal waters are among the most productive ecosystems in the world and provide shelter and nursery grounds to many species, including important commercial species of fish, crustaceans and cephalopods [12–14].

Among shallow-water habitats, tide pools are probably the best sentinels to assess the impacts of global warming on marine assemblages. Tide pools add important niche space to coastal habitats and commonly host diverse biological communities [15]. They often support important macroalgal canopy [16, 17] that creates a tridimensional environment with abundant shelter and food resources for small organisms that are in turn prey to secondary consumers [18, 19]. While using the tide pool environment, however, small invertebrates and fish find refuge from most predators, since low depth excludes large consumers. Due to these favourable conditions, tide pools are often nursery areas for larvae and juveniles of marine fish and shrimp [20–22]. However, these small water bodies have much lower thermal inertia than surrounding nearshore waters, occasionally attaining exceedingly high temperatures, mostly during summer ebb-tide periods and especially in higher tide pools [23]. Very fast variation of air temperature can lead to lethal conditions and may result in mass mortality of many different species, as repeatedly observed in tropical shores [23–25]. Natural selection of behavioral and other physiological traits may not catch up with the ongoing trend of increased warming, leading to maladaptive selection of tide-pool habitats [26–29]. This scenario is consistent to the ecological trap concept, initially coined for maladaptive nesting bird strategies [1], and verified for a wide range of taxa [2, 3], including the well-known case of insect attraction to polarized light causing death upon contact [2].

Heat-waves occur when the daily maximum temperature of more than five consecutive days exceeds the average maximum temperature by 5°C (the average maximum being estimated for the period 1961–1990). They are predicted to increase in intensity, duration and frequency as a consequence of climate change. However, most studies on the effects of warming on living organisms focus on mean temperatures and fail to address the potential impacts of the predicted change in the severity and the temporal occurrence of heat waves [30]. Since tide pool waters are very sensitive to heat waves, it becomes important to study the role of tide pools as ecological traps. If heat-wave induced mortality events are already observed at tide pools [24, 25], it is reasonable to assume they will become more frequent in the future due to climate warming. However, heat waves can also lead to acclimatization of the upper thermal limits of tide pool dwellers. If organisms undergo acclimatization prior to being trapped in tide pools, their survival chances may increase.

Also relevant is the ongoing debate on whether tropical species are more vulnerable to climate warming than temperate species. This has important implications for conservation priorities at a global scale. The rate of climate warming is predicted to be lower in the tropics than in temperate zones [31, 32]. However, species that evolved in more thermally stable environments, like the tropics, may suffer disproportionately from small increases in temperature, while species that live in strongly seasonal environments, like temperate zones, prone to wider temperature variation, may tolerate greater temperature shifts [33–36]. This way, tropical organisms would be more vulnerable to future warming than their temperate counterparts [33]. Although tolerating higher temperatures, tropical species exhibit lowest thermal plasticity, as predicted by the “trade-off hypothesis” [37–39]. Moreover, several studies have shown that more tolerant species are actually living closer to their upper thermal limits [23, 40, 41].

Thermal vulnerability will ultimately depend on the organisms’ thermal window, acclimation response and genetic adaptation potential [42], which remain unknown for most species. Evaluating the effects of heat waves on tropical and temperate species, particularly on whether warming episodes elicit an increase of their thermal limits, and by how much, is a critical task for a more realistic assessment of the impact of global warming on coastal marine organisms. In the present work, we set out to test if an increase in temperature, consistent with that attained by subtidal waters during heat waves (10 days at +3°C = “heat wave experiment”) [43, 44] can elicit an increase in the upper thermal limits of tropical and temperate coastal organisms. We aimed to test realistic temperatures and we used a dynamic method, the critical thermal maximum, which mimics the natural thermal ramp that occurs in tide pools in summer [23], to determine the upper thermal limits of these organisms, before and after the “heat wave experiment”. Finally, we compared the temperatures attained at natural tide pools and the upper thermal limits of the organisms tested to verify if (1) tide pools could be considered ecological traps, and whether (2) the increase in upper thermal limits elicited by the “heat wave experiment” could acclimate tested organisms, reducing their vulnerability to further heat stress. The effects of region (tropical vs temperate) and taxonomic group (mollusks, crustaceans and fish) on the acclimation response, and both the average and the among-individual variation of upper thermal limits (respectively indicating tolerance and potential for local selection), were tested.

## Material and methods

The authors declare that the experiments followed the Portuguese and Brazilian legislation for animal experimentation. Ethics committees in Portugal and Brazil specifically authorized this experiment. Authorization document 0421/000/000/2013 from the Portuguese authorities (DGAV) and 13.1.981.53.7 from the Brazilian authorities (CEUA, USP—Ribeirão Preto).

### Study areas

**Fig 1:**
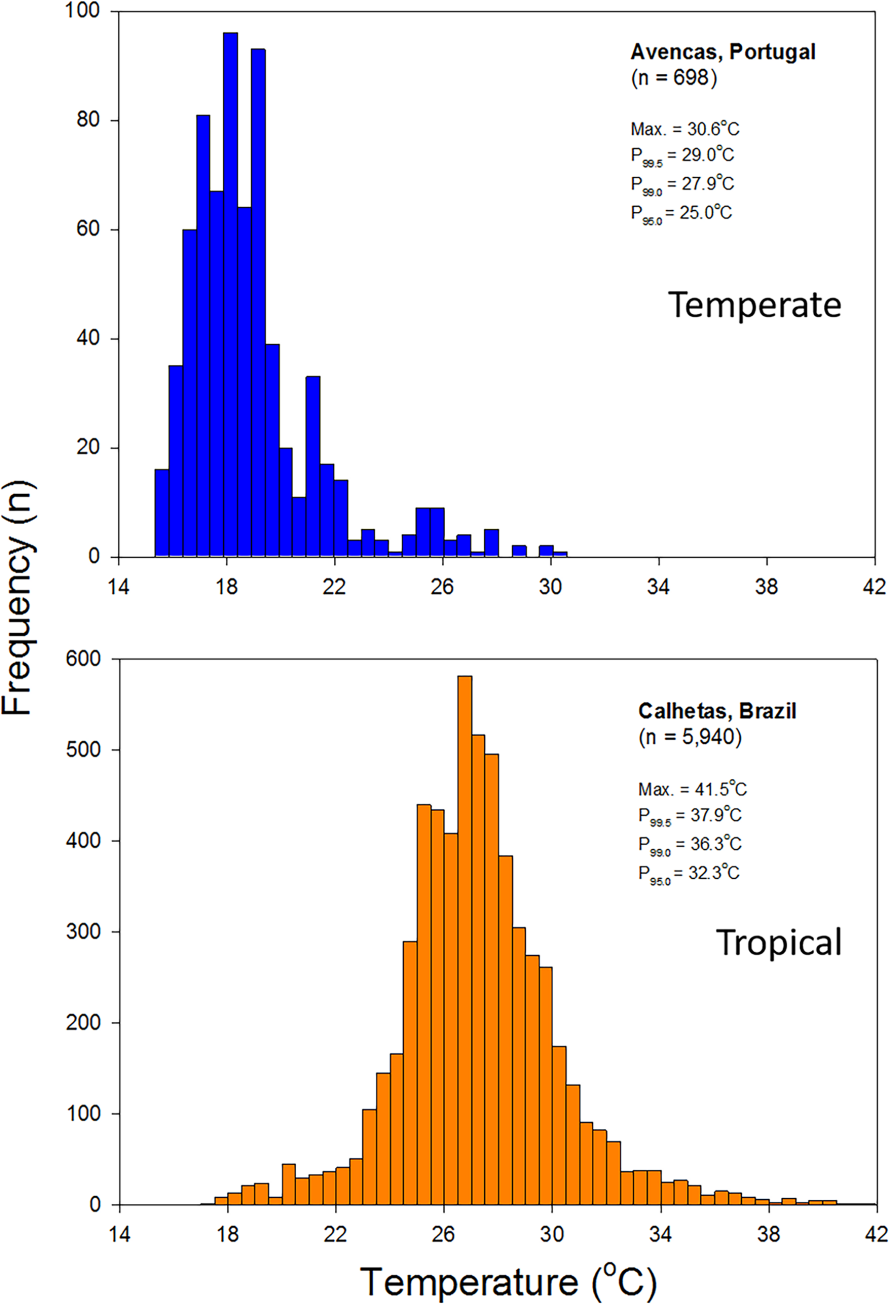
Overall temperature frequency distributions at tide-pools continuously monitored at Avencas (Portugal) and Calhetas (Brazil) during summer. Data from Avencas was obtained from six replicate pools; two sampled at 15 min intervals in 2014 over 4 d and four sampled hourly in 2015 over 2d. Data from Calhetas was obtained from six replicate pools sampled continuously over 56 d at 2 h intervals during the summer of 2015/6.

Mollusks, crustaceans and fish were collected in a tropical and a temperate coastal area, in Southeastern Brazil (São Sebastião–Ubatuba, São Paulo State) and Central Portugal Portugal (Cascais–Avencas), respectively, in the summer of 2015. Two sites, distanced approximately 70 km, were chosen in each area for the sampling of organisms (23^o^49’S; 45^o^25’W and 23°27’S; 45°03’W in Brazil and 38^o^41′ N; 9^o^21′ W and 38° 26’N; 8°50’W in Portugal). Tides are semidiurnal in both areas. All the tide pools selected for this study (seven pools in each area) were located in the lower intertidal, where a higher diversity of species occurs. Similarly sized pools were selected, with a mean depth of 0.3 m and a maximum depth of 0.5 m. All species examined in this study make extensive use of lower tide pools. In order to assess whether or not tide-pools may act as ecological traps in the sampled regions, temperature datasets obtained using Onset Hobo V2 and Maxim iButtons probes deployed at the bottom of random tide pools during the summer of 2014 and 2015 at Cascais-Avencas (summer 2014, 2015, Central Portugal), and during the summer of 2016 at Calhetas (São Sebastião, Brazil), were examined. First, we compared equivalent datasets at specific periods from just a few days before (3–6 d) to the end of the warmest season in central Portugal (maximum daily averages over 26°C, from June 19 to September 22) and the warmest season in São Paulo, Brazil (over 27°C, from January 3 to March 20), according to historical data (weatherspark.com). Then we compiled all available data for a more precise estimation of extreme temperature percentiles (P_99.5_, P_99.0, P95_), which unlike maximum temperature records are not affected by sample size (Fig 1).

### Species tested

In total 46 coastal species were tested, 23 tropical species and 23 temperate species. Both transient and resident species were tested. The tropical species studied were the mollusks *Lottia subrugosa* (d'Orbigny, 1846), *Morula nodulosa* (C.B. Adams, 1845), *Echinolittorina lineolata* (d'Orbigny, 1840), *Stramonita haemastoma* (Linnaeus, 1767), *Strombus pugilis* Linnaeus, 1758, *Tegula viridula* (Gmelin, 1791); the crustaceans *Callinectes danae* Smith, 1869, *Clibanarius antillensis* Stimpson, 1859, *Epialtus brasiliensis* Dana, 1852, *Eriphia gonagra* (Fabricius, 1781), *Eurypanopeus abbreviatus* (Stimpson 1860), *Menippe nodifrons* Stimpson 1859, *Microphrys bicornutus* (Latreille, 1825), *Pachygrapsus transversus* (Gibbes 1850), *Pagurus brevidactylus* (Stimpson, 1859), *Palaemon northropi* (Rankin 1898), *Panopeus americanus* Saussure, 1857; and the teleost fish *Abudefduf saxatilis* (Linnaeus, 1758), *Bathygobius soporator* (Valenciennes 1837), *Diplodus argenteus* (Valenciennes, 1830), *Eucinostomus melanopterus* (Bleeker 1863), *Odontesthes argentinensis* (Valenciennes, 1835) and *Scartella cristata* (Linnaeus 1758).

The temperate species studied were the mollusks *Acanthochitona crinita* (Pennant, 1777), *Calliostoma zizyphinum* (Linnaeus, 1758), *Cerithium vulgatum* Bruguière, 1792, *Gibbula umbilicalis* (da Costa, 1778), *Lepidochitona cinerea* (Linnaeus, 1767), *Melarhaphe neritoides* (Linnaeus, 1758), *Nassarius reticulatus* (Linnaeus, 1758), *Ocenebra erinaceus* (Linnaeus, 1758), *Phorcus lineatus* (da Costa, 1778), *Mytilus galloprovincialis* Lamarck, 1819; the crustaceans *Pagurus prideaux* Leach, 1815, *Pagurus bernhardus* (Linnaeus, 1758), *Palaemon elegans* (Rathke 1837), *Palaemon serratus* (Pennant 1777), *Pirimela denticulata* (Montagu, 1808), *Lophozozymus incisus* (Milne-Edwards 1834); and the teleost fish *Coryphoblennius galerita* (Linnaeus, 1758), *Diplodus sargus* (Linnaeus, 1758), *Gobius paganellus* Linnaeus, 1758, *Lepadogaster lepadogaster* (Bonnaterre, 1788), *Lipophrys pholis* (Linnaeus, 1758), *Pomatoschistus microps* (Krøyer 1838) and *Syngnathus acus* Linnaeus, 1758.

### Acclimation conditions and critical thermal maxima

The organisms were collected in tide pools, by hand or with hand-nets, and transported to the laboratory. They were kept in closed-system aquaria with constant temperature, aerated sea water and salinity of 35‰. The dissolved O_2_ level varied between 95% and 100%. Each individual aquarium was 25 x 25 x 25 cm. The individuals of each species were randomly placed in two aquaria, species were separated to avoid the additional stress of inter-species agonistic relations and/or predator-prey behavior. Organisms were fed daily *ad libitum* and starved for 24h prior to temperature trials. Fish, crabs and shrimp were fed with frozen shrimp muscle and commercial fish pellets (commercial brand Continente, Portugal). Omnivorous and herbivorous mollusks were given natural rocks covered with the macroalgae *Ulva* sp. and commercial fish pellets.

Organisms were kept for seven days at the same temperature as the habitat temperature found in the natural environment at the time of capture, 29.0°C (±0.5°C) for tropical organisms and 22.0°C (±0.5°C) for temperate ones, to ensure that all had a similar recent thermal history and minimal thermal disturbance. At the end of this 7-day period, the critical thermal maximum (CTMax) was determined for a subset of these organisms to determine control values of CTMax (CTMax_control_).

Afterwards, organisms were acclimated for 10 days at 3°C above the acclimation temperature, 32°C for tropical organisms and 25°C for temperate ones, mimicking a heat wave period in nature [43, 44]. At the end of this 10 day-period, CTMax was estimated for another subset of individuals of each species (CTMax_10 days_). Although the experiment was sequential, different organisms of each species were tested in each CTMax trial, i.e. no organism was exposed to more than one CTMax trial (all individuals that were subjected to a CTMax trial were excluded from the remaining experiment).

The CTMax method is a widely used dynamic method of quantifying the upper thermal limits of ectothermic vertebrates and invertebrates [23, 45–49]. It is determined by exposing the organisms to a constant thermal ramp until a critical point is reached (e.g. loss of balance [50–52]). [46] defined CTMax as the “arithmetic mean of the collective thermal points at which the end-point is reached”, the end-point being loss of equilibrium. In shrimp and fish, loss of equilibrium was defined as the point when individuals could not swim straight and started moving in an angled position. Crabs were forced upside down with tweezers, and an end-point was recorded if they were unable to get back upright. These criteria are the same followed by [53] and [23, 54]. Gastropods were placed in a transparent container and allowed to attach to its walls and move around, inactive specimens were discarded [55]. Every 10 min, containers were tipped over to identify which organisms could remain attached and which had reached CTMax, by losing their attachment [56]. Bivalves were continuously observed and CTMax was determined when they opened their valves and relaxed their foot muscle, simultaneously.

All organisms were subjected to a thermostatic bath with a constant rate of water-temperature increase of 1°C/15 min, with constant aeration and observation, until they reached the end-point. This temperature ramp is consistent to what can be found in tide pools during summer days, following the recommendations of [57] for the use of ecologically realistic warming ramps. The experiments were carried out in shaded day light (14 L; 10D). The temperature at which each animal reached its end-point was measured with a digital thermometer and registered.

The total length of all individuals was measured at the end of the CTMax experiment (Table 1). Fish were measured with an ichthyometer (total length) and shrimps (total length), crabs (maximum carapace width) and mollusks (maximum shell length) with a digital slide caliper. Sample sizes were similar to those used by [46], [53] and [23, 54] (Table 1).

**Table 1:** Taxonomic group, sample size and mean length of the individuals used to estimate the CTMax_control_ and the CTMax_10 days_.

		CTMax_control_			CTMax_10 days_		
	Taxonomic group	Sample size	Length (mm)	s.d.	Sample size	Length (mm)	s.d.
**Tropical species**							
*Lottia subrugosa*	Mollusca	19	13.4	3.2	20	13.0	1.9
*Morula nodulosa*	Mollusca	29	16.0	2.9	21	17.8	1.8
*Echinolittorina lineolata*	Mollusca	15	3.8	0.9	14	3.9	0.9
*Stramonita haemastoma*	Mollusca	10	31.4	8.3	15	28.7	6.3
*Strombus pugilis*	Mollusca	13	62.8	16.2	5	72.2	2.2
*Tegula viridula*	Mollusca	15	23.1	1.1	10	21.2	2.3
*Callinectes danae*	Crustacea	9	36.8	19.2	10	58.1	8.5
*Clibanarius antillensis*	Crustacea	16	25.4	5.6	7	29.1	6.5
*Epialtus brasiliensis*	Crustacea	15	5.3	1.1	19	5.1	0.8
*Eriphia gonagra*	Crustacea	7	28.1	2.7	7	31.8	6.2
*Eurypanopeus abbreviatus*	Crustacea	64	15.3	2.9	70	17.5	3.9
*Menippe nodifrons*	Crustacea	39	24.9	7.6	48	22.0	7.1
*Microphrys bicornutus*	Crustacea	5	13.6	4.2	6	6.0	1.4
*Pachygrapsus transversus*	Crustacea	32	10.8	3.9	33	13.7	4.3
*Pagurus brevidactylus*	Crustacea	12	18.4	4.8	10	15.7	2.7
*Palaemon northropi*	Crustacea	55	24.7	7.6	57	25.2	4.6
*Panopeus americanus*	Crustacea	17	7.8	2.3	12	8.3	1.3
*Abudefduf saxatilis*	Fish	16	22.1	5.6	15	23.8	2.9
*Bathygobius soporator*	Fish	14	36.1	15.4	19	31.0	11.9
*Diplodus argenteus*	Fish	7	44.1	8.4	12	45.5	11.3
*Eucinostomus melanopterus*	Fish	12	30.3	8.6	7	29.8	6.3
*Odontesthes argentinensis*	Fish	11	35.2	12.0	7	45.5	12.0
*Scartella cristata*	Fish	25	41.7	11.5	19	42.2	16.9
**Temperate species**							
*Acantochitona crinita*	Mollusca	7	9.1	1.7	5	13.0	2.4
*Calliostoma zizyphinum*	Mollusca	6	16.2	3.9	9	13.0	1.8
*Cerithium vulgatum*	Mollusca	9	35.0	3.8	9	34.6	4.4
*Gibbula umbilicalis*	Mollusca	10	11.7	2.6	6	10.8	1.0
*Lepidochitona cinerea*	Mollusca	10	10.0	2.8	12	7.8	2.0
*Melarhaphe neritoides*	Mollusca	34	3.2	0.7	23	3.3	0.6
*Nassarius reticulatus*	Mollusca	20	19.6	3.1	25	17.3	4.4
*Ocenebra erinaceus*	Mollusca	10	24.2	2.7	13	23.8	2.9
*Phorcus lineatus*	Mollusca	19	14.2	2.4	17	12.4	1.7
*Mytilus galloprovincialis*	Mollusca	92	19.5	6.1	85	21.2	5.2
*Pagurus prideaux*	Crustacea	11	26.6	8.3	18	25.8	6.3
*Pagurus bernhardus*	Crustacea	5	21.0	5.6	7	22.8	8.0
*Palaemon elegans*	Crustacea	37	27.0	7.6	37	26.9	5.7
*Palaemon serratus*	Crustacea	26	30.8	9.0	41	34.1	11.4
*Pirimela denticulata*	Crustacea	5	15.2	1.6	5	15.6	1.2
*Lophozozymus incisus*	Crustacea	11	22.2	7.2	10	17.9	3.5
*Coryphoblennius galerita*	Fish	22	23.9	4.6	27	27.5	7.1
*Diplodus sargus*	Fish	10	44.8	11.2	14	39.1	8.7
*Gobius paganellus*	Fish	10	42.6	8.0	12	45.9	9.3
*Lepadogaster lepadogaster*	Fish	10	61.7	16.2	19	57.4	11.4
*Lipophrys pholis*	Fish	9	52.8	9.3	7	42.4	6.8
*Pomatoschistus microps*	Fish	16	28.4	3.1	18	27.7	3.4
*Syngnathus acus*	Fish	6	106.0	11.6	5	70.0	33.6

s.d. stands for standard deviation.

### Data analyses

#### CTMax

The upper thermal limits for each species were calculated using the equation:

$${\mathrm{CTMax}}_{\left(\mathrm{species}\right)}=\sum \left(T_{\mathrm{end}-\mathrm{point}\;n}\right)/n$$

Where T_end-point_ is the temperature at which the end-point was reached for any given individual, and n stands for sample size.

To estimate intraspecific variability of the CTMax, the 95% confidence interval was estimated for each species.

### Acclimation response

The acclimation response was defined as the difference between the CTMax after warming (CTMax_10days_) and the CTMax registered after the control period (CTMax_control_).

### Statistical analyses

T-tests were conducted to investigate if CTMax_control_ values were different from the CTMax_10days_ values for each species. Factorial analyses of variance (ANOVA) were conducted to test the effect of region (tropical *vs* temperate) and taxonomic group (Mollusca *vs* Crustacea *vs* Teleostea) in CTMax_control_, acclimation response and 95% confidence interval (of the CTMax_control_), using estimates measured of each species as replicates. The effect of tide pool was also tested in a similar way. Prior to these tests, normality and homoscedasticity were confirmed.

The phylogenetic independent contrasts method was used in a linear-regression analysis between CTMax_-control_ and acclimation response to test the “trade-off” hypothesis. Tests were performed using the software COMPARE, version 4.6b [58], including a nested, hierarchical random term representing taxonomic affinities of the taxa (phylum/class/order/family/genus/spp) to account for non-independence of data, since no phylogenetic tree is available for all taxa included in this study.

## Results

**Fig 2:**
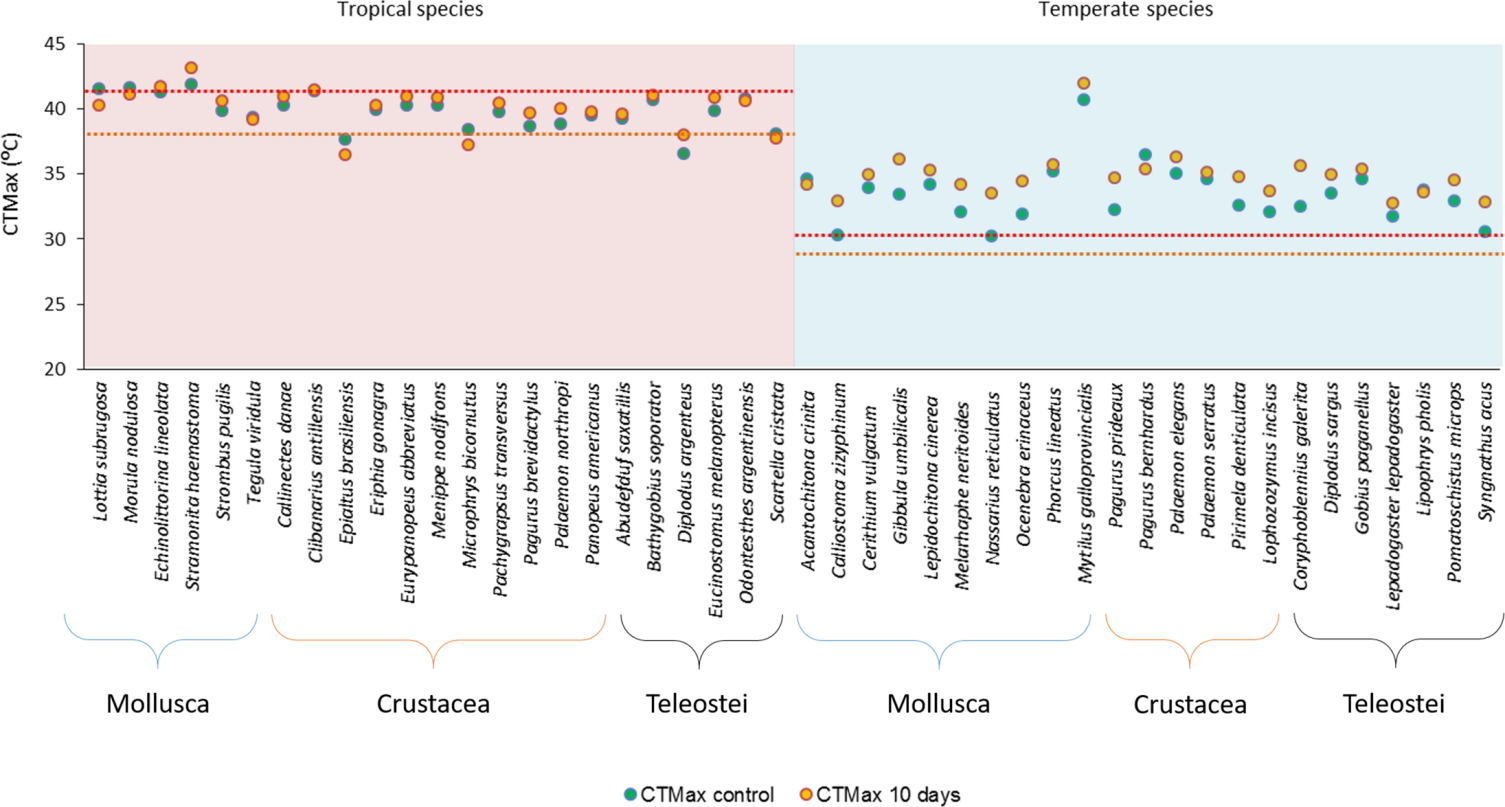
Critical thermal maximum (CTMax) of the control (CTMax_control_), in green dots, and after 10 days at a +3°C temperature (CTMax_10 days_), in orange dots, for each species. Tropical species are presented in the red background area, while temperate species are presented in the blue background area. The red dotted line indicates the highest recorded water temperature in tropical (41.5°C) and temperate (30.6°C) tide pools. The orange dotted line indicates the percentile 99.5 of water temperature in tropical (37.9°C) and temperate (29.0°C) tide pools (Fig 1).

**Fig 3:**
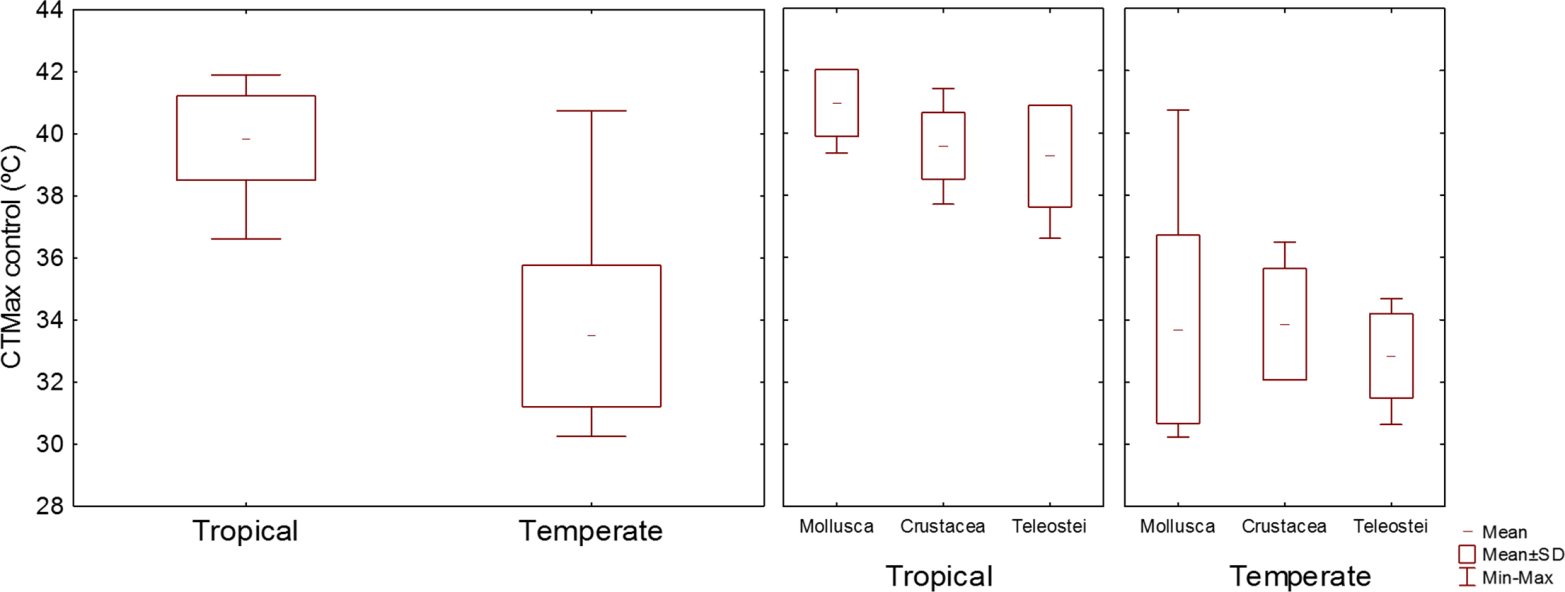
Distribution of the values of CTMax_control_ of tropical and temperate organisms.

The highest CTMax_control_ was that of the tropical mollusk *S*. *haemastoma*, 41.90°C, while the highest CTMax_control_ for temperate species was that of the mollusk *M*. *galloprovincialis*, 40.74°C (Fig 2). The mean CTMax_control_ for tropical species was 39.86°C, while for temperate species it was 33.48°C (Fig 3). The tropical species with the lowest CTMax_control_, 36.61°C, was the fish *D*. *argenteus*, while the lowest CTMax_control_ of temperate species was 30.24°C for the mollusk *N*. *reticulatus* (Fig 2).

**Fig 4:**
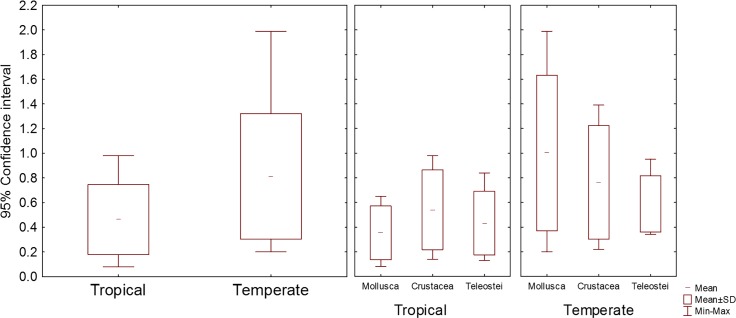
Confidence intervals (95%) of CTMax control values of tropical and temperate organisms.

The tropical species with the highest acclimation response, 1.40°C, was *D*. *argenteus*, while the temperate species with the highest acclimation response, 3.35°C, was *N*. *reticulatus*, meaning that the species with the lowest CTMax_control_ were the ones with the highest acclimation response, both for tropical and temperate species (Figs 2 and 4).

**Fig 5:**
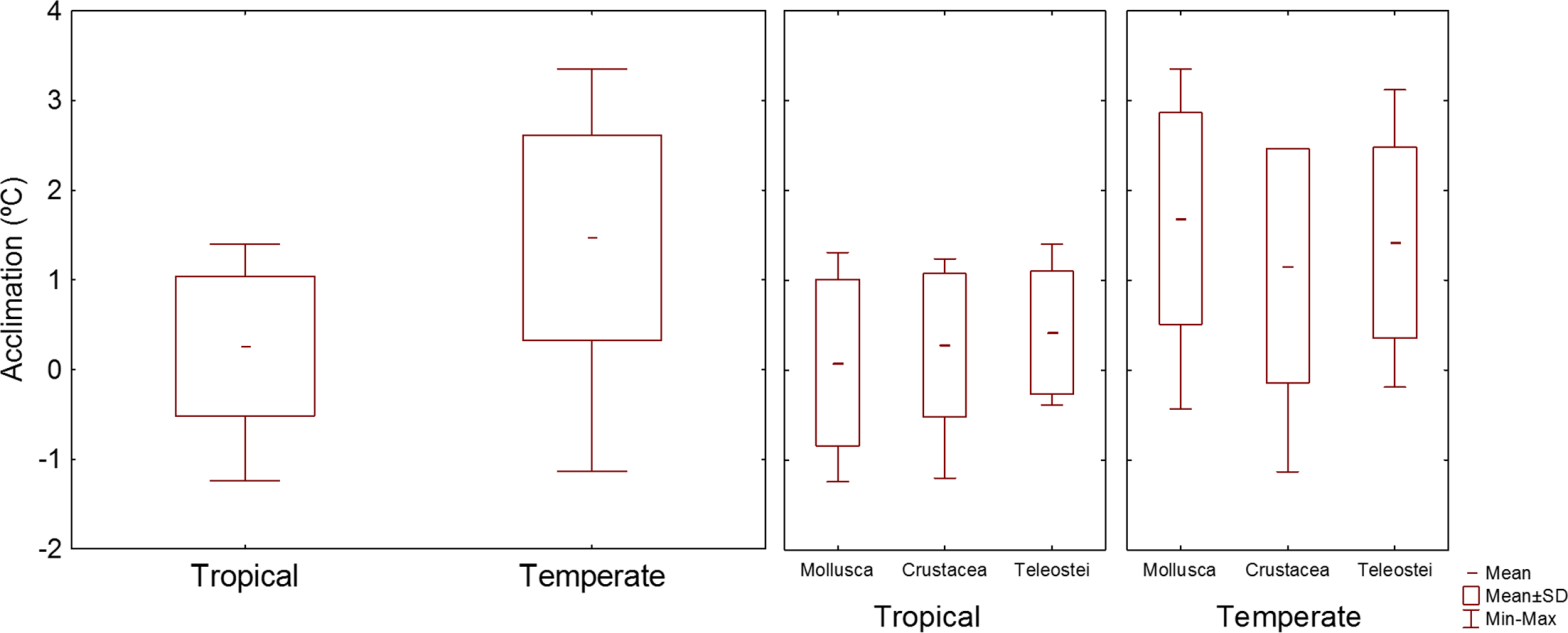
Distribution of the values of acclimation response of tropical and temperate organisms.

**Fig 6:**
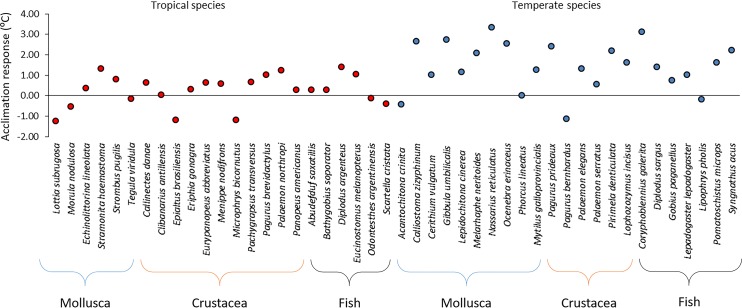
Acclimation response (CTMax_10 days_—CTMax_control_) of tropical (in red) and temperate (in blue) coastal species (values different from zero have a p<0.05).

Of the 23 tropical species tested, 13 could not acclimate, while for the 23 temperate species tested, only 5 did not acclimate (Figs 5 and 6). The mean acclimation response of the tropical species was 0.38°C, while for the temperate species it was 1.47°C (Figs 4 and 5). The mean 95% confidence interval of the CTMax_control_ values was 0.5 for tropical organisms and 0.8 for temperates organisms (Fig 4). A negative significant correlation was found between acclimation response and CTMax_control_, with acclimation response decreasing with increasing CTMax_control_ (r^2^ = 0.42, P < 0.05).

The effect of region (tropical *vs* temperate) was significant for CTMax_control_, 95% confidence interval and acclimation response (Table 2). The effect of taxonomic group (Mollusca *vs* Crustacea *vs* Teleostei) was not significant for any of the variables tested (Table 2). The effect of tide pool was not significant (p > 0.01).

**Table 2:** ANOVA results for the effect of latitude (tropical *vs* temperate) and taxonomic group (Mollusca *vs* Crustacea *vs* Fish) on CTMax_control_, 95% confidence interval (CI) and acclimation.

		CTMax_control_								
		Average			95% CI			Acclimation		
	df	MS	F	p-value	MS	F	p-value	MS	F	p-value
Region:R	1	450.9	128.9	**<0.0001**	1.26	7.6	**<0.001**	12.5	16	**<0.0001**
Taxon: T	2	5.6	1.6	0.212	0.1	0.7	0.513	0	0	0.956
RXT	2	2.3	0.7	0.529	0.3	1.6	0.220	0.1	0.1	0.926
Residual	40	3.5			0.2			0.8		

Significant results are presented in bold.

Among tropical species, only 3 out of 23 species presented a CTMax_control_ above the maximum habitat temperature (MHT = 41.5°C) and only 2 species had a CTMax_10days_ above MHT, rendering tide pool as effective ecological traps in the tropics when temperature reaches MHT (Fig 2). This was remarkably different for temperate species since 21 out of the 23 species tested presented a CTMax_control_ above MHT (30.6°C) and all species had a CTMax_10days_ above MHT (Fig 2). When the percentile 99.5 of water temperature is used as a reference, 21 out of 23 tropical species have CTMax_control_ values above it and 20 out of 23 have a CTMax_10days_ above it, while all temperate species tested present a CTMax_control_ and a CTMax_10days_ well above this reference temperature (Fig 2).

## Discussion

This work shows, for the first time, the role of tide pools as ecological traps in tropical coastal areas. Mortality in tropical tide pools had been previously reported by [24], which observed that the surgeon fish *Acanthurus triostegus* and the blenny *Istiblennius edentulous* died when tide pool water temperature reached 37°C. However, no study had previously tried to thoroughly test the upper thermal limits of the most common species that occur in tide pools and compare them to the extreme temperatures that tide pool waters can attain.

In this study it became evident that the vast majority of the tropical species tested had upper thermal limits below the maximum habitat temperature (MHT) of tide pools (Figs 1 and 2), with the exception of three gastropods, the limpet *L*. *subrugosa* and the sea snails, *M*. *nodulosa* and *S*. *haemastoma*. Additionally, it was shown that the acclimation response of such organisms, after a short-term period at +3°C, was very small or non-existent (Figs 2 and 6). In fact, two of the three species that had an upper thermal limit above the MHT, *L*. *subrugosa* and the sea snails, *M*. *nodulosa*, presented a decrease in upper thermal limit, after the acclimation period, that placed them below the MHT. Leaving *S*. *haemastoma* as the only tropical species with an upper thermal limit above MHT and that was also able to increase that upper thermal limit, by 1.31°C, after the acclimation period (Figs 2 and 6).

It can thus be concluded that tropical tide pools are ecological traps, where most of the species tested risk not surviving during the ebb tide in warm summer days and heat waves. This has the potential to result in important population losses in tropical coastal areas where tide pools are abundant. It also incurs important problems for recruitment, since many of the organisms that use tide pools as refuge, are juveniles. [20, 21] suggested that tide pools are alternative nursery areas for coastal fish, given the high densities of larvae and juveniles of some marine fishes found in tide pools. In the present study, all of the tropical fish collected in tide pools and tested were juveniles (Table 1). All of the five tropical fish species studied had upper thermal limits below the MHT and only two species, *D*. *argenteus* and *E*. *melanopterus*, showed some acclimation response, albeit not enough to increase their upper thermal limits above the MHT (Fig 2). When these fish juveniles stay in tide pools during the warmest summer days, they are likely to not survive and thus will not contribute to the adult stocks of their species. As climate warming progresses and mean and extreme temperatures increase, the ecological trap effect of tropical tide pools is likely to increase even more with potentially severe effects for the population sizes of the common coastal species that get trapped in them. This gives support to the hypothesis that tropical species will suffer disproportionally from small increases in temperature, because they are already living very close to their thermal limits [23, 33–35, 40, 41].

The present work also gives an important contribution to the debate on whether the tropical or temperate species are more vulnerable to climate warming [33, 59], since it shows that the ecological trap effect of tide pools is likely to be severe in the tropics but not relevant in temperate areas. In fact, the results from the present study indicate that tide pools are probably not ecological traps in temperate areas, since their maximum water temperature is well below the upper thermal limits of most of the organisms tested, meaning that temperate organisms have a considerable thermal safety margin in these environments (Figs 1 and 2). It also shows that after a short period of time (10 days) at only +3°C, the majority of temperate organisms tested were able to increase their upper thermal limits well above the maximum temperature attained by tide pool waters (Figs 1 and 2). This shows that temperate organisms seem well equipped to face todays’ thermal extremes and also future higher temperatures.

Furthermore, although the upper thermal limits of tropical organisms were significantly higher than those of temperate organisms, the mean acclimation response of the tropical species was significantly lower than that of temperate organisms. Also, the mean of the 95% confidence interval (of the CTMax_control_) of the tropical species was much lower than that of temperate species. The low intraspecific variation in the upper thermal limits exhibited by tropical species in comparison to that of temperate species indicates that such species have lower phenotypic variation suggesting a lower evolutionary potential to deal with current and future warming via the process of natural selection [34].

Other authors had previously reported that species adapted to high temperatures, with very high upper limits, seemed to have done so at the expense of the phenotypic plasticity of those limits, thus revealing an evolutionary trade-off between upper thermal limits and acclimation response [42, 60]. In addition, their upper thermal limits are very close to the maximum habitat temperature, meaning that their safety margin towards naturally occurring extreme temperature is reduced [23, 42, 61–63].

The data from the present study support the trade-off hypothesis. Not only was a negative correlation found between CTMax_control_ and acclimation response, with acclimation response decreasing with increasing CTMax_control_, but interestingly, the species with the lowest CTMax_control_, *D*. *argenteus* among the tropical species and *N*. *reticulatus* among the temperate species, were also the ones with the highest acclimation response. The most important difference between these two species being that *D*. *argenteus* upper thermal limit was still below the MHT after the acclimation, while the upper thermal limit of *N*. *reticulatus* was well above the MHT after the acclimation, meaning that for the temperate species acclimation is an effective way of increasing survival chances in tide pools, but it is not for the tropical species.

The results from the present study largely corroborate those reported by [23], which studied the thermal limits of 35 subtidal and intertidal coastal species and the acclimation response of 8 intertidal species in a long-term experiment, at the same study areas. They concluded that all species had some acclimation response with the exception of the two gobid fish tested, *B*. *soporator* (tropical) and *P*. *microps* (temperate). However, in the present study the temperate gobid *P*. *microps* as well as *Gobius paganellus* did acclimate, indicating that thermal tolerance of gobiid fish populations may change in time.

[23] found that the taxonomic group (decapod crustaceans *vs* teleost fish) did not have an effect on the thermal limit, nor on the acclimation response of the organisms tested. The present work, which tested mollusks, crustaceans and teleost fish, came to a similar conclusion. Similar studies with amphibians [64] and insects [61] also concluded that warming in the tropics will have more deleterious consequences than in the temperate zones. [61] pointed out that the data available for several invertebrate and vertebrate taxa suggested that this is a general pattern for terrestrial ectotherms. The results from the present study, along with those from [23], provide a strong indication that such general pattern can be extended to coastal marine ectotherms.

Underlying this general pattern is certainly a physiological process that sets the thermal limits of ectotherms. [65] put forward the idea that there is a unifying principle among metazoans, where the borders of the thermal tolerance window are characterized by the onset of internal systemic hypoxia despite fully oxygenated waters, resulting in anaerobic metabolism that cannot be sustained for long periods of time [65–67]. This phenomena along with the general exponential increase in metabolism with warming found for ectotherms [68–70] probably drives the observed latitudinal patterns in thermal vulnerability.

In conclusion, the present work showed that tropical tide pools are ecological traps for an important number of common coastal species, given that they can attain temperatures considerably higher than their upper thermal limits. Tide pools are not ecological traps in temperate zones because their maximum temperatures are well below the upper thermal limits of most of the common tide pool species. Although tropical species have higher thermal limits than temperate species, they have lower acclimation response, which does not allow them to increase their upper thermal limits above the maximum habitat temperature of tide pools, when exposed to an acclimation period of 10 days at +3°C. This way, tropical coastal organisms seem to be not only more vulnerable to climate warming *per se*, but also to an increase in the ecological trap effect of tide pools. This effect could have important consequences for recruitment of juveniles to the adult stocks of subtidal organisms that use tide pools during their juvenile phase.

However, it must be considered that more prolonged, frequent and stronger acclimation periods, produced by natural heat waves, may elicit a stronger acclimation response than that observed in this study. Also, epigenetic inheritance may result in transgenerational acclimation to higher temperatures. Organisms surviving extreme heat waves and breeding thereafter may produce a progeny with higher thermal limits, as has been shown in the tropical fish *Achanthochromis polyacanthus* [71]. Future studies that replicate future environmental conditions and their effects over the present and future generations of organisms should elucidate the differential effect of climate warming on tropical and temperate coastal organisms and the potential deleterious effects of tide pools.

## References

[1] Dwernychuk LW, Boag DA. Ducks nesting in association with gulls—an ecological trap? Can J Zool. 1972; 50: 559–563.

[2] Kriska G, Horvath G, Andrikovics S. Why do mayflies lay their eggs en masse on dry asphalt roads? Water-imitating polarized light reflected from asphalt attracts Ephemeroptera. J Exp Biol. 1998; 201: 2273–2286. 9662498 10.1242/jeb.201.15.2273

[3] Battin J. When good animals love bad habitats: ecological traps and the conservation of animal populations. Conserv Biol. 2004; 18: 1482–1491.

[4] Helmuth B, Moore P, Mieszkowska N, Hawkins SJ. Living on the Edge of Two Changing Worlds: Forecasting the Responses of Rocky Intertidal Ecosystems to Climate Change. Ann Rev Ecol Evol Syst. 2006; 37: 373–404.

[5] Menge B. Response of a rocky intertidal ecosystem engineer and community dominant to climate change. Ecol Lett. 2008; 11: 151–162. doi: 10.1111/j.1461-0248.2007.01135.x 18034837 10.1111/j.1461-0248.2007.01135.x

[6] Firth LB, Crowe TP, Moore P, Thompson R, Hawkins S. Predicting impacts of climate-induced range expansion: an experimental framework and a test involving key grazers on temperate rocky shores. Glob Change Biol. 2009; 15: 1413–1422.

[7] Firth LB, White FJ, Schofield M, Hanley ME, Burrows MT, Thompson RC, et al Facing the future: the importance of substratum features for ecological engineering of artificial habitats in the rocky intertidal. Mar Fresh Res. 2016; 67: 131–143.

[8] Hawkins SJ, Sugden HE, Mieszkowska N, Moore PJ, Poloczanska E, Leaper R, et al Consequences of climate-driven biodiversity changes for ecosystem functioning of North European rocky shores. Mar Ecol Progr Ser. 2009; 396: 245–259.

[9] Rastrick SPS, Calosi P, Calder-Potts R, Foggo A, Nightingale G, Widdicombe S, et al Living in warmer, more acidic oceans retards physiological recovery from tidal emersion in the velvet swimming crab, *Necora puber*. J Exp Biol. 2014; 217: 2499–2508. doi: 10.1242/jeb.089011 24803457 10.1242/jeb.089011

[10] Morley SA, Btes AR, Lamare M, Richard J, Nguyen KD, Brown J, et al Rates of warming and the global sensitivity of shallow water marine invertebrates to elevated temperature. J Mar Biol Ass UK. 2014; 96: 1–7.

[11] Collard M, Rastrick SPS, Calosi P, Demolder Y, Dille J, Findlay HS, et al The impact of ocean acidification and warming on the skeletal mechanical properties of the sea urchin *Paracentrotus lividus* from laboratory and field observations. ICES J Mar Sci. 2016; 73: 727–738.

[12] Lasiak TA. Nursery grounds of juvenile teleosts: evidence from the surf zone of King’s Beach, Port Elizabeth. S African J Sci. 1981; 77: 388–390.

[13] Nagelkerken I, Velde G, Gorissen MW, Meijer GJ, Van’t Hof T, den Hartoga C. Importance of Mangroves, Seagrass Beds and the Shallow Coral Reef as a Nursery for Important Coral Reef Fishes, Using a Visual Census Technique. Estuar Coast Shelf Sci. 2000; 51: 31–44.

[14] Amara R, Paul C. Seasonal patterns in the fish and epibenthic crustaceans community of an intertidal zone with particular reference to the population dynamics of plaice and brown shrimp. Estuar Coast Shelf Sci. 2003; 56: 807–818.

[15] Firth LB, Schofield M, White FJ, Skov MW, Hawkins SJ. Biodiversity in intertidal rock pools: Informing engineering criteria for artificial habitat enhancement in the built environment. Mar Environ Res. 2014; 102: 122–130. doi: 10.1016/j.marenvres.2014.03.016 24746927 10.1016/j.marenvres.2014.03.016

[16] Dethier MN. Pattern and process in tidepool algae: factors influencing seasonality and distribution. Botan Mar. 1982; 25: 55–66.

[17] Dethier MN. Disturbance and recovery in intertidal pools: maintenance of mosaic patterns. Ecol Monogr. 1984; 54: 99–118.

[18] Trussell GC, Ewanchuck PJ, Bertness MD, Silliman BR. Trophic cascades in rocky shore tide pools: distinguishing lethal and nonlethal effects. Oecologia. 2004; 139: 427–432. doi: 10.1007/s00442-004-1512-8 14872337 10.1007/s00442-004-1512-8

[19] Kruck NC, Chargulaf CA, Saint-Paul U, Tibbets TR. Early post-settlement habitat and diet shifts and the nursery function of tidepools during *Sillago* spp. recruitment in Moreton Bay, Australia. Mar Ecol Progr Ser. 2009; 384: 207–219.

[20] Dias M, Cabral HN, Silva A, Vinagre C. Diet of marine fish larvae and juveniles that use rocky intertidal pools at the Portuguese coast. J App Ichthyol. 2014; 30: 970–977.

[21] Dias M, Roma J, Fonseca C, Pinto M, Cabral HN, Silva A et al Intertidal pools as alternative nursery habitats for coastal fishes. Mar Biol Res. 2016; 12: 331–344.

[22] Vinagre C, Dias M, Fonseca C, Pinto MT, Cabral H, Silva A. Use of rocky intertidal pools by shrimp species in a temperate area. Biologia. 2015a; 70: 372–379.

[23] Vinagre C, Leal I, Mendonça V, Madeira D, Narciso L, Diniz MS, et al Vulnerability to climate warming and acclimation capacity of tropical and temperate coastal organisms. Ecol Ind. 2016; 62: 317–327.

[24] Hiatt RW, Strasburg DW. Ecological relationships of the fish fauna on coral reefs of the Marshall Islands. Ecol Monogr. 1960; 30: 65–127.

[25] Little C, Williams GA, Trowbridge CD. The biology of rocky shores. Oxford University Press, UK; 2009 pp. 356.

[26] Schlaepfer MA, Runge MC, Sherman PW. Ecological and evolutionary traps. Tre Ecol Evol. 2002; 17: 474–480.

[27] Robertson BA, Hutto RL. A framework for understanding ecological traps and an evaluation of existing evidence. Ecology. 2006; 87: 1075–1085. 16761584 10.1890/0012-9658(2006)87[1075:affuet]2.0.co;2

[28] Robertson BA, Rehage JS, Sih A. Ecological novelty and the emergence of evolutionary traps. Tr Ecol Evol. 2013; 28: 552–560. 10.1016/j.tree.2013.04.004 23756104

[29] Hale R, Treml EA, Swearer SE. Evaluating the metapopulation consequences of ecological traps. Proc Royal Soc B. 2015; 282: 20142930. 10.1098/rspb.2014.2930 PMC4375870 25761712

[30] Sorte CJB, Fuller A, Bracken ES. Impacts of a simulated heat wave on composition of a marine community. Oikos. 2010; 119: 1909–1918.

[31] IPCC. Climate Change 2007: Synthesis Report. Fourth Assessment Report of the Intergovernmental Panel on Climate Change. Pachauri RK, Reisinger A editors, IPCC, Geneva, Switzerland; 2007 pp. 104.

[32] IPCC. Climate Change 2013: The Physical Science Basis. Contribution of Working Group I to the Fifth Assessment Report of the Intergovernmental Panel on Climate Change (Stocker TF, Qin D, Plattner GK, Tignor M, Allen SK, Boschung J, et al. editors) Cambridge University Press, Cambridge, United Kingdom and New York, NY, USA; 2013.

[33] Tewksbury JJ, Huey RB, Deutsch CA. Putting the Heat on Tropical Animals. Ecology. 2008; 320: 1296–1297. 10.1126/science.1159328 18535231

[34] Hoffmann GE, Todgham AE. Living in the Now: Physiological Mechanisms to Tolerate a Rapidly Changing Environment. Ann Rev Physiol. 2010; 72: 127–145. 20148670 10.1146/annurev-physiol-021909-135900

[35] Pörtner HO, Peck MA. Climate change effects on fishes and fisheries: towards a cause-and-effect understanding. J Fish Biol. 2010; 77: 1745–1779. doi: 10.1111/j.1095-8649.2010.02783.x 21078088 10.1111/j.1095-8649.2010.02783.x

[36] Nguyen KDT, Morley SA, Lai C, Clark MS, Tan KS, Bates AE, et al Upper temperature limits of tropical marine ectotherms: global warming implications. PLoS ONE. 2011; 6: 1–8. 10.1371/journal.pone.0029340 PMC3248430 22242115

[37] Chown SL. Physiological variation in insects: hierarchical levels and implications. J Insect Physiol. 2001; 47: 649–660. 11356411 10.1016/s0022-1910(00)00163-3

[38] Overgaard J, Kristensen TN, Mitchell KA, Hoffmann AA. Thermal tolerance in widespread and tropical Drosophila species: does phenotypic plasticity increase with latitude? Am Natural. 2011; 178: S80– S96. 10.1086/661780 21956094

[39] Gunderson AR, Stillman JH. Plasticity in thermal tolerance has limited potential to buffer ectotherms from global warming. Proc Royal Soc B. 2015; 282: 1471–2954. 10.1098/rspb.2015.0401 PMC4455808 25994676

[40] Somero GN. Comparative physiology: a “crystal ball” for predicting consequences of global change. Am J Physiol. 2011; 301: R1–R14. 10.1152/ajpregu.00719.2010 21430078

[41] Jost JA, Podolski SM, Frederich M. Enhancing thermal tolerance by eliminating the pejus range: a comparative study with three decapod crustaceans. Mar Ecol Progr Ser. 2012; 444: 263–274.

[42] Stillman JH. Acclimation Capacity Underlies Susceptibility to Climate Change. Science. 2003; 301, 65 doi: 10.1126/science.1083073 12843385 10.1126/science.1083073

[43] Garrabou J, Coma R, Bensoussan N, Bally M, Chevaldonné P, Cigliano M, Diaz D, Harmelin JG, Gambi MC, Kersting DK, Ledoux JB, Lejeusne C, Linares C, Marschal C, Pérez T, Ribes M, Romano JC, Serrano E, Teixido N, Torrents O, Zabala M, Zuberer F Cerrano C. Mass mortality in Northwestern Mediterranean rocky benthic communities: effects of the 2003 heat wave. Global Change Biology. 2009 15; 1090–1103.

[44] Meehl GA et al Global climate projections–In: SolomonS. et al (eds), Climate change 2007: the physical science basis. Contribution of Working Group I to the 4th Assessment Rep. of the Intergovernmental Panel on Climate Change. Cambridge Univ. Press 2007; pp. 747–845.

[45] Cuculescu M, Hyde D, Bowler K. Thermal Tolerance of Two Species of Marine Crab, *Cancer pagurus* and *Carcinus maenas*. J Thermal Biol. 1998; 23: 107–110.

[46] Mora C, Ospina A. Tolerance to high temperatures and potential impact of sea warming on reef fishes of Gorgona Island (tropical eastern Pacific). Mar Biol. 2001; 139: 765–769.

[47] Salas A, Díaz F, Re AD, Glindosanchez CE, Sanchez-Castrejon E, González M, et al Preferred Temperature, Thermal Tolerance, and Metabolic Response of *Tegula regina* (Stearns, 1892). J Shellfish Res. 2014; 33: 239–246.

[48] Kaspari M, Clay NA, Lucas J, Yanoviak SP, Kay A. Thermal adaptation generates a diversity of thermal limits in a rainforest ant community. Glob Change Biol. 2015; 21: 1092:1102. 10.1111/gcb.12750 25242246

[49] Regil JN, Mascaro M, Díaz F, Re AD, Sánchez-Zamora A, Caamal-Monsreal C, et al Thermal biology of prey (*Melongena corona bispinosa*, *Strombus pugilis*, *Callinectes similis*, *Libinia dubia*) and predators (*Ocyurus chrysurus*, *Centropomus undecimalis*) of *Octopus maya* from the Yucatan Peninsula. J Thermal Biol. 2015; 53: 151–161. 10.1016/j.jtherbio.2015.11.001 26590468

[50] Brattstrom BH. Thermal acclimation in Anuran amphibians as a function of latitude and altitude. Comp Biochem Phys. 1968; 24: 93–111. 10.1016/0010-406x(68)90961-4 5689525

[51] Huey RB, Crill WD, Kingsolver JG, Weber KE. A method for rapid measurement of heat or cold resistance of small insects. Func Ecol. 1992; 6: 489–494.

[52] Lutterschmidt W.I. and Hutchison V.H., 1997. The critical thermal maximum: history and critique. Can J Zool. 1997; 75: 1561–1574.

[53] Madeira D, Narciso L, Cabral H, Vinagre C. Thermal tolerance and potential impacts of climate change on coastal and estuarine organisms. J Sea Res. 2012; 70: 32–41.

[54] Vinagre C, Dias M, Roma J, Silva A, Madeira D, Diniz M. Critical thermal maxima of common rocky intertidal fish and shrimps–a preliminary assessment. J Sea Res. 2013; 81: 10–12.

[55] Clarke AP. The nature of heat coma in *Littorina littorea* (Mollusca: Gastropoda). Mar Biol. 2000; 137: 447–451.

[56] Sorte CJB, Hofmann GE. Thermotolerance and heat-shock protein expression in Northeastern Pacific *Nucella* species with different biogeographical ranges. Mar Biol. 2005; 146: 985–993.

[57] Vinagre C, Leal I, Mendonça V, Flores AV. Effect of warming rates on the Critical Thermal Maxima of fish, crabs and shrimp. J Thermal Biol. 2015b; 47: 19–25. 10.1016/j.jtherbio.2014.10.012 25526650

[58] Martins E P. COMPARE, version 4.6b. 2004 Computer programs for the statistical analysis of comparative data Distributed by the author at http://compare.bio.indiana.edu/. Department of Biology, Indiana University, Bloomington IN.

[59] Ghalambor CK. Are mountain passes higher in the tropics? Integr Comp Biol. 2006; 46: 5–17. doi: 10.1093/icb/icj003 21672718 10.1093/icb/icj003

[60] Angilletta MJ, Wilson RS, Navas CA, James RS. Tradeoffs and the evolution of thermal reaction norms. Trends Ecol Evol. 2003; 18: 234–240.

[61] Deutsch CA, Tewsbury JJ, Huey RB, Sheldon KS, Ghalambor CK, Haak DC, et al Impacts of climate warming on terrestrial ectotherms across latitude. Proc Nat Acad Sci USA. 2008; 105: 6668–6672. doi: 10.1073/pnas.0709472105 18458348 10.1073/pnas.0709472105 PMC2373333

[62] Diederich CM, Pechenik JA. Thermal tolerance of *Crepidula fornicata* (Gastropoda) life history stages from intertidal and subtidal subpopulations. Mar Ecol Progr Ser. 2013; 486: 173–187.

[63] Overgaard J, Kearney MR, Hoffmann AA. Sensitivity to thermal extremes in Australian Drosophila implies similar impacts of climate change on the distribution of widespread and tropical species. Glob Change Biol. 2014; 20: 1738–1750. 10.1111/gcb.12521 24549716

[64] Duarte H, Tejedo M, Katzenberger M. Can amphibians take the heat? Vulnerability to climate warming in subtropical and temperate larval amphibian communities. Glob Change Biol. 2012; 18: 412–421.

[65] Pörtner HO. Climate change and temperature dependent biogeography: systemic to molecular hierarchies of thermal tolerance in animals. Comp Biochem Physiol A. 2002; 132: 739–761. 10.1016/s1095-6433(02)00045-4 12095860

[66] Pörtner HO. Climate change and temperature dependent biogeography: oxygen limitation of thermal tolerance in animals. Naturwissenschaft. 2001; 88: 137–146. 10.1007/s001140100216 11480701

[67] Pörtner HO, Knust R. Climate change affects marine fishes through the oxygen limitation of thermal tolerance. Science. 2007; 315: 95–97. doi: 10.1126/science.1135471 17204649 10.1126/science.1135471

[68] Brown JH, Gillooly JF, Allen AP, Savage VM, West GB. Towards a metabolic theory of ecology. Ecology. 2004; 85: 1771–1789.

[69] Glazier DS. A unifying explanation for diverse metabolic scaling in animals and plants. Biol Rev. 2010; 85: 111–13. doi: 10.1111/j.1469-185X.2009.00095.x 19895606 10.1111/j.1469-185X.2009.00095.x

[70] Vucic-Pestic O, Ehnes R, Rall BC, Brose U. Warming up the system: higher predator feeding rates but lower energetic efficiencies. Glob Change Biol. 2011; 17: 1301–1310.

[71] Donelson JM, Munday PL, Mccormick MI, Nilsson GE. Acclimation to predicted ocean warming through developmental plasticity in a tropical reef fish. Glob Change Biol. 2012; 17: 1712–1719.

